# Magnetic properties of nitrogen-doped ZrO_2_: Theoretical evidence of absence of room temperature ferromagnetism

**DOI:** 10.1038/srep31435

**Published:** 2016-08-16

**Authors:** Elisa Albanese, Mirko Leccese, Cristiana Di Valentin, Gianfranco Pacchioni

**Affiliations:** 1 Dipartimento di Scienza dei Materiali, Università Milano Bicocca, via R. Cozzi 55, 20125 Milano, Italy

## Abstract

N-dopants in bulk monoclinic ZrO_2_ and their magnetic interactions have been investigated by DFT calculations, using the B3LYP hybrid functional. The electronic and magnetic properties of the paramagnetic N species, substitutionals and interstitials, are discussed. Their thermodynamic stability has been estimated as a function of the oxygen partial pressure. At 300 K, N prefers interstitial sites at any range of oxygen pressure, while at higher temperatures (700–1000 K), oxygen poor-conditions facilitate substitutional dopants. We have considered the interaction of two N defects in various positions in order to investigate the possible occurrence of ferromagnetic ordering. A very small magnetic coupling constant has been calculated for several 2N-ZrO_2_ configurations, thus demonstrating that magnetic ordering can be achieved only at very low temperatures, well below liquid nitrogen. Furthermore, when N atoms replace O at different sites, resulting in slightly different positions of the corresponding N 2p levels, a direct charge transfer can occur between the two dopants with consequent quenching of the magnetic moment. Another mechanism that contributes to the quenching of the N magnetic moments is the interplay with oxygen vacancies. These effects contribute to reduce the concentration of magnetic impurities, thus limiting the possibility to establish magnetic ordering.

Zirconium dioxide (ZrO_2_) is an interesting material used in several technological applications. Pure ZrO_2_ can exist in three main polymorphs: i) monoclinic (m-ZrO_2_) (*T* < 1480 K); ii) tetragonal (t-ZrO_2_) (1480 < *T* < 2650 K) and iii) cubic (c-ZrO_2_) (*T* > 2650 K)^1^,^2^. The cubic phase has a fluorite structure, while the other two derive from progressive distortions of the cubic one. The monoclinic polymorph is stable at low temperature, but it has scarce applications in ceramic technologies. In contrast, the two high temperature phases (cubic and tetragonal) have excellent mechanical, thermal and dielectric properties, which make ZrO_2_ an ideal candidate for protective coatings, high-k dielectric materials, chemically inert refractory materials, etc. The room temperature stabilization of these polymorphs is commonly achieved by transition metal doping, such as Ce^4+^, Y^3+^ and Ga^3+^. ZrO_2_ is widely used as a solid electrolyte in oxygen sensors^3^ and in solid oxide fuel cells (SOFCs) (Y- or Ca-doped ZrO_2_)^4^, nuclear waste confinement^5^ and as a gate dielectric material in metal-oxide semiconductor devices^6^. It is also an important material in heterogeneous catalysis and, to a lesser extent, in photocatalysis. The interest in ZrO_2_ for photocatalysis is limited by both the large band-gap energy (≈5 eV) and the weak activity in photo-oxidation, although it has been recently demonstrated that Ce-doped ZrO_2_ is a photoactive material under visible light^7^.

The inclusion of a nitrogen dopant into the oxide lattice is a credible approach to tune the absorption properties either by narrowing the band gap or by introduction of new energy states into the gap, as demonstrated for other oxides^8^,^9^,^10^. In addition, the insertion of 2p light elements (e.g. C and N) into oxide semiconductors has recently attracted a lot of interest because of their potential applications as diluted magnetic semiconductor (DMS) both in spintronics and opto-electronics, such as light emitting devices, detectors, ultra low power memory device, etc.

In the last decade, many efforts have been dedicated to the study of ferromagnetism induced by transition metal (TM) doping of semiconductor oxides. Many TM-doped systems have been reported and predicted as ferromagnets, such as ZnO^11^,^12^,^13^, TiO_2_^14^,^15^,^16^ and ZrO_2_^17^,^18^. In particular, several reports indicate that pure ZrO_2_ exhibits room temperature ferromagnetism and that this is related to the presence of oxygen vacancies or structural defects. Some of these studies also show that the crystallographic phase is very important in this context, with reports of ferromagnetism more common for tetragonal ZrO_2_ structures^18^,^19^,^20^. However, this field has continued to be dogged by concerns regarding clustering problem and second ferromagnetic phases formation^21^,^22^. The work of Kenmochi *et al*. on the d^0^ ferromagnetism in semiconductor oxides^23^,^24^,^25^,^26^ has pioneered the strategy to introduce 2p light elements to achieve room temperature ferromagnetism (RTFM) and to overcome some of these problems. Both experimentally and theoretically, ferromagnetism has been recently proposed for C- and N-ZnO^27^,^28^, C- and N-TiO_2_^29^,^30^,^31^,^32^ and N-In_2_O_3_^33^. Furthermore, a recent theoretical work of H. Zhu *et al*.^34^. predicted p-ferromagnetism behavior for N-ZrO_2_.

In spite of the large number of publications of the last years, the nature and the origin of the ferromagnetism in doped semiconductors are often controversial. Moreover, most if not all the calculations regarding this research field were performed at general gradient approximation (GGA) level of theory, which is well known to give an inaccurate description of the oxide electronic structure, in particular for the energy band gap description and localization of magnetic states^35^.

To shed light on the nature of the p-ferromagnetism in N-doped oxides, we focused on one of the most interesting and promising RTFM, N-ZrO_2_, by using a hybrid functional (B3LYP), i.e. a self-interaction corrected functional which is particularly appropriate to better describe the band gap and the spin properties of solid systems. In fact, self-interaction corrected (SIC) DFT functionals, such as SIC-LDA, have been successfully adopted in the past, showing remarkable improvement in the description of highly correlated electron systems. The LDA and GGA functionals often overestimate the hybridization between electron states due to the underestimation of the band gap energies of the semiconductors^36^,^37^,^38^.

A systematic study of several possible doped structures has therefore been performed and the corresponding magnetic configurations were investigated. In this way, the interaction between two isolated paramagnetic centers has been theoretically evaluated in terms of effective pair exchange interaction. Finally, the expected Curie temperature for the transition from anti-ferro to ferromagnetic phases has been estimated by means of Heisenberg mean field model. We will show that room temperature p-ferromagnetism in N-ZrO_2_ is definitely ruled out, for various reasons.

The paper is organized as follows. In the next section we concisely illustrate the theoretical methods used for the calculations and other computational details. In the Results and Discussion section we report the main outcomes of the present work by discussing (i) the structure and the electronic properties of singly doped N-ZrO_2_; (ii) the hyperfine coupling constants of N-ZrO_2_ and (iii) the simulation of several possible double doped 2N-ZrO_2_ structures, their electronic and magnetic properties. Summary and Conclusions are reported in the last section.

## Computational Details

The investigation of N-doped ZrO_2_ was carried out with periodic DFT calculations employing the Becke-3^39^ and Lee-Yang-Parr^40^ (B3LYP) exchange and correlation functional as implemented in the CRYSTAL14 program^41^. Hybrid functionals are indeed known to provide a much more robust description of spin-polarized systems than the GGA approximation^42^,^43^. The choice to use B3LYP instead of other, theoretically more sophisticated, hybrid functionals is dictated by the fact that using the same approach we have studied in the past other N-doped oxides: N-TiO_2_, N-SnO_2_, N-ZnO, N-MgO^44^,^45^. This allows us to directly compare the nature of a Nitrogen impurity in oxides with different electronic and geometric structure, thus providing the basis for a solid comparative study.

Crystalline orbitals are represented as linear combinations of Bloch functions (BF) and are evaluated over a regular three-dimensions mesh of points in reciprocal space. Each BF is built from local atomic orbitals (AO) resulting from contractions (i.e. linear combinations with constant coefficients) of Gaussian-type-functions which in turn are the product of a Gaussian times a real solid spherical harmonic function. All electron basis set have been used for O and N atoms: 8–411(d1) and 7–311(d1), respectively. A 311(d31) basis set associated with the Hay and Wadt small-core effective core potential (ECP) was used for the Zr atom. The computed Kohn-Sham band gap in Γ is 5.73 eV (minimum band gap 5.30 eV). This is higher than the measured bandgap of 4.2 eV using electron energy loss spectroscopy (EELS)^46^, but it is within the range measured using vacuum ultraviolet (VUV) absorption spectroscopy (5.78–6.62 eV)^47^. For the numerical integration of exchange-correlation term, 75 radial points and 974 angular points (XLGRID) in a Lebedev scheme in the region of chemical interest were adopted. The Pack-Monkhorst/Gilat shrinking factors for the reciprocal space were set to 6 for the ZrO_2_ pure system and to 4 for the N-ZrO_2_ structures, corresponding respectively to 40 and 36 real reciprocal space points at which the Hamiltonian matrix was diagonalized.

The calculations of the N-ZrO_2_ structures were performed on a 96 atoms supercell, corresponding to a 2 × 2 × 2 unit cell of monoclinic ZrO_2_. The optimized cell parameters of the non-defective system are consistent with the experimental values (see Supporting Information). The accuracy of the integral calculations was increased with respect to its default value by setting the tolerances to 7, 7, 7, 7 and 18. The self-consistent field (SCF) iterative procedure converged to a tolerance in total energy of ΔE = 1 · 10^−7^ a.u. The above computational parameters ensured a full numerical convergence on all the computed properties described in this work. All the crystal structures are fully optimized (i.e. both cell parameters and internal coordinates) without symmetry operators in order to allow a complete structural relaxation. However, the cell parameters of the ZrO_2_ are not much affected by the N inclusion. The differences of the doped cells with respect to the undoped one are, indeed, always below 0.2% (see Supporting Information).

The hyperfine spin-Hamiltonian, H_hfc_ = **S** · **A** · **I**, is given in terms of the hyperfine matrix **A**, which described the coupling of the electron with the nuclear spin.

## Results and Discussion

### N-doped ZrO_2_: Structure and Electronic Properties

**Figure 1:**
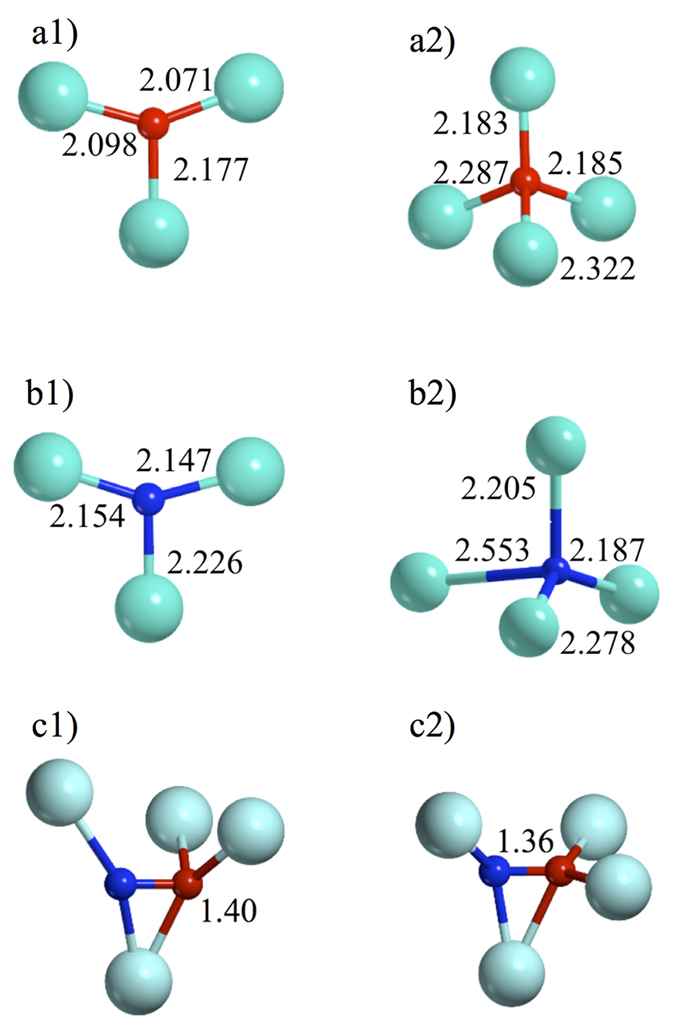
Local structural features of pure ZrO_2_ (a1, a2) and the four N-doped ZrO_2_ structure. (**b1**) N_sub3c_; (**b2**) N_sub4c_; (**c1**) N_int3c_ and (**c2**) N_int4c_. Selected bond lengths (Å) are reported.

Four different N-doped ZrO_2_ models have been simulated, two where N is substitutional to oxygen and two where N is in an interstitial site. In the ZrO_2_ monoclinic cell, two different oxygens are present. The lattice oxygen can, indeed, be bound to three Zr atoms, O_3c_, or to four Zr atoms, O_4c_. Therefore, the N dopant can form N_sub3c_-ZrO_2_ and N_sub4c_-ZrO_2_ structures. The two interstitial structures are obtained by adding an N atom into the supercell in proximity to an O_3c_ (N_int3c_-ZrO_2_) and an O_4c_ (N_int4c_-ZrO_2_), respectively (see Fig. 1). As reported for other oxide systems, the substitution of an O atom with N (both O_3c_ and O_4c_) does not affect the geometry, showing negligible changes in the cell parameters and local structure. On the contrary, the inclusion of an interstitial N atom leads to a remarkable rearrangement of the atomic structure with the spontaneous formation of a N—O species with three electrons localized in the π antibonding orbitals^44^,^45^. The N−O bond lengths, 1.40 and 1.36 Å for N_int3c_ and N_int4c_, respectively, are very close to those found in N-TiO_2_ (d(N–O) = 1.36 Å)^48^.

**Figure 2:**
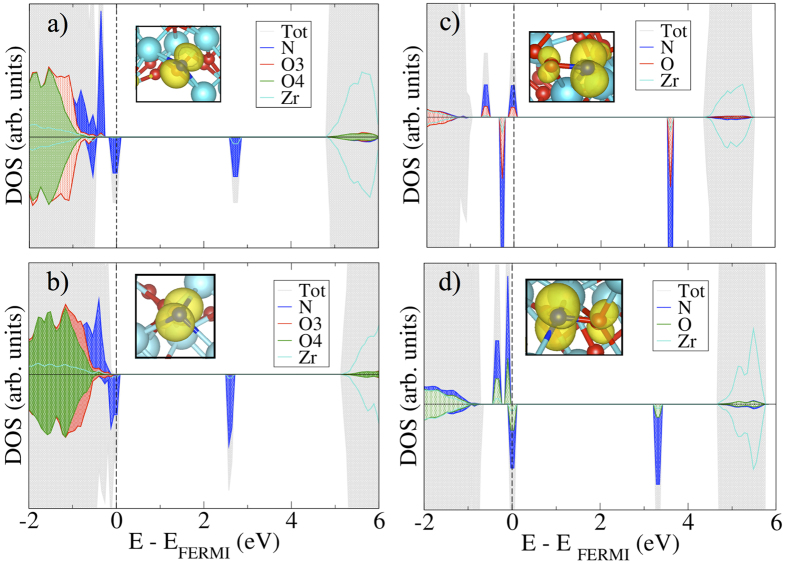
Total and Projected Densities of States of the N-doped structures. (**a**) N_sub3c_ -ZrO_2_; (**b**) N_sub4c_ -ZrO_2_; (**c**) N_int3c_ -ZrO_2_ and (**d**) N_int4c_ -ZrO_2_. Grey line represents TDOS, blue N atom, light blue Zr, red and green O_3c_ and O_4c_, respectively. The insets show the spin density plots (isodensity threshold values 0.007). The Fermi level is set to the highest occupied level (dashed line).

The computed total and projected densities of states (TDOS and PDOS) for N, and the neighboring Zr and O ions are shown in Fig. 2. N_sub_ introduces a singly occupied N 2p_α_ state, which lies above the O 2p valence band (VB) in the N_sub3c_-ZrO_2_ and below the top of VB in N_sub4c_-ZrO_2_. This different behavior is due to the higher coordination sphere of the N_sub4c_ that stabilizes the N states, thus lowering their energy. In the tri-coordinated dopant, this state is fully localized on the N atom, while in N_sub4c_, some hybridization between N 2p and O 2p band states is observed. The corresponding empty 2p_β_ component (hole state), instead, is fully localized on the N 2p state for both substitutional structures and lies about 2.0–2.4 eV below the bottom of the CB (Fig. 2). In both cases, the spin densities are fully localized on the N 2p state (ρ = 0.92 and 0.86 for N_sub3c_ and N_sub4c_, respectively).

N_int_ species are associated with a singly occupied π molecular orbital, which lies just above the top of the VB (Fig. 2). The corresponding empty components are very high in the band gap. Also in the interstitial case, the N_4c_ states are slightly more stable than the N_3c_ ones. The large energy separation between the filled and empty components of the impurity state indicates a strong exchange splitting which is typical of highly localized unpaired electrons. In this case, the unpaired electron is shared between the N and O atoms of the NO species (Fig. 2); the spin densities are localized on the π system, thus reducing the localization to 0.84 and 0.80 for N_int3c_ and N_int4c_, respectively.

If we consider the relative stabilities of the different models, N_sub3c_-ZrO_2_ results slightly more stable than N_sub4c_ by about 0.2 eV, while the difference between the two interstitial sites is much more pronounced, with N_int3c_ being more stable by 1.2 eV.

In order to compare the overall stability of the two types of doped systems (substitutional vs interstitial), we have to consider the following reactions:


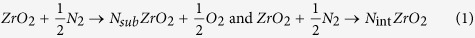


then we obtain


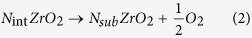


The relative stabilities of these species vary as a function of the oxygen chemical potential (*μ*_o_) that characterizes the oxygen environment during the synthesis. The environment acts as a reservoir, which can give or take any amount of oxygen without changing its temperature and pressure^49^,^50^. Oxygen-poor conditions correspond to a low value of *μ*_*O*_, and, conversely, oxygen-rich conditions correspond to a high value of *μ*_O_. The formation free energy of this reaction at given temperature and pressure is defined as:


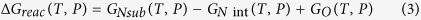


where G_Nsub_ and G_Nint_ are the Gibbs free energies of N_sub_-ZrO_2_ and N_int_-ZrO_2_, respectively, and G_O_ is the free energy of an O atom.

Assuming that the free energy of the doped systems can be approximated by the electronic energy, i.e.:


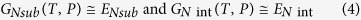


The Gibbs free energy of an oxygen atom, G_O_(T, P), is given by:


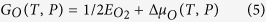


where the change in oxygen chemical potential with pressure and temperature, Δμ_O_(T, P), is defined as:


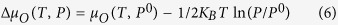


The standard pressure P^0^ is defined as 1 atm, while the values of standard chemical potential, μ_O_ (T, P^0^), at 300 K, 700 K and 1000 K are taken from the work of Reuter and Scheffler^49^.

**Figure 3:**
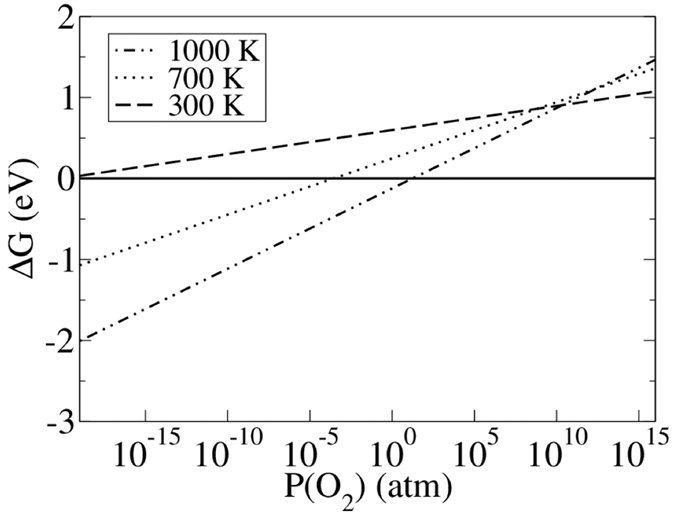
N_int3c_-ZrO_2_/N_sub3c_-ZrO_2_ stability phase diagram as a function of O_2_ partial pressure at 300 K, 700 K and 1000 K in terms of the reaction (2), i.e. N_int_ZrO_2_ --> N_sub_ZrO_2_ + 1/2 O_2_. The solid line represents ΔG = 0 eV.

In Fig. 3, we report the stability phase diagram of the most stable configurations, N_int3c_–ZrO_2_ and N_sub3c_–ZrO_2_, as a function of oxygen pressure at room and higher temperatures (i.e. 300 K, 700 K and 1000 K). It results that, at 300 K, N is incorporated in interstitial sites at any range of oxygen pressure. This is expected because of the formation of the new bonds due to the inclusion of an interstitial atom. At higher temperatures, typical of the annealing process in the synthesis of oxides, such as C- and N- TiO_2_^43^,^51^, the oxygen poor-conditions (below p(O_2_) = 10^−3^ atm at 700 K) are more favorable for introducing substitutional dopants.

### N-doped ZrO_2_: EPR Properties and Magnetic Ordering

#### Hyperfine Coupling Constants

Electron paramagnetic resonance is a powerful technique to identify isolated magnetic impurities in bulk materials. Several examples of the successful combined use of theory and experiment to fully identify the nature of N-dopants in semiconducting and insulating oxides have been reported in the past^43^,^52^. So far, no reports of EPR spectra of N-ZrO_2_ seems to exist in the literature. Here we report the computed hyperfine coupling constants and the spin densities for the various sites considered (see Table 1). In the substitutional structures, the spin densities are fully localized on the N 2p state, as depicted in Fig. 2 (ρ = 0.92 and 0.86 for N_sub3c_ and N_sub4c_, respectively). On the contrary, in the interstitial systems, the unpaired electron is localized on the π molecular N—O orbital and, therefore, shows lower ρ values, as observed for other N-doped oxides.

The ^14^N hyperfine coupling constants are slightly different in the interstitial and substitutional structures. In particular, the a_iso_ term, which corresponds to the Fermi contact term (proportional to the electron spin density in the nuclear volume), is expected to be positive in the N-centered radical species and is almost double in N_int_ with respect to the N_sub_ configurations (0.85 mT vs 0.4 mT). The dipolar matrix **T** has the typical form of the electron–nucleus dipolar interaction for an electron in a p orbital (i.e. 2T, -T, -T); the T values are quite similar for all the N-doped ZrO_2_ species, a fact that is common also to other cases of N-doped oxides considered in the past^44^,^45^.

#### Magnetic Coupling

**Figure 4:**
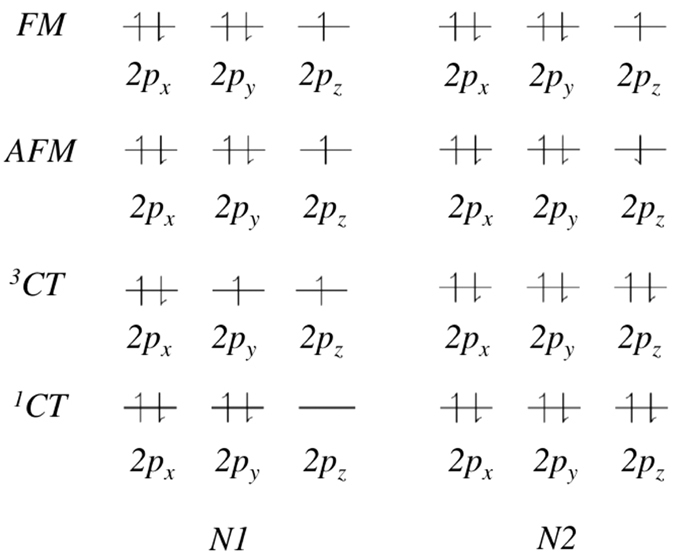
Scheme of the electron configurations considered.

In order to determine the existence of p-ferromagnetism in N-doped ZrO_2_, two lattice oxygen atoms have been replaced by two N dopants in the 2 × 2 × 2 supercell. Considering the above N_int3c_-ZrO_2_/N_sub3c_-ZrO_2_ phase diagram, substitutional dopants are taken into account. The structures correspond to a doping concentration of 3.1%, consistent with that commonly achieved in the experiments. The following possible models have been explored: i) 2N_sub3c_-ZrO_2_; ii) N_sub3c_/N_sub4c_-ZrO_2_ and iii) 2N_sub4c_-ZrO_2_. The two N atoms are separated by about 4 Å. For each case, the ferromagnetic (FM) and antiferromagnetic (AFM) configurations have been considered as a starting solution. However, as it will be discussed in more detail below, other electronic configurations are possible. In particular, for the N_sub3c_/N_sub4c_-ZrO_2_ and the 2N_sub4c_-ZrO_2_ models, an internal charge transfer (CT) between two N impurities can take place and, therefore, other two configurations has been considered. One N-dopant acts as a donor and the other as an acceptor with formal change of their oxidation states. The donor changes its configuration from N 2p^5^ to N 2p^4^ giving rise to two possible spin configurations, triplet ^3^CT or singlet ^1^CT. The N acceptor changes its configuration from 2p^5^ to 2p^6^ and therefore does not contribute to the magnetic structure. The corresponding electron configurations are depicted in Fig. 4.

The different models are discussed in the next subsections.

2N_sub3c_-ZrO_2_: Effective Pair Exchange Coupling: In the first model, 2N_sub3c_-ZrO_2_, two N atoms replace two O_3c_ atoms at the distance of 3.8 Å. The optimization of the internal coordinates does not affect the N-N distance (the variation is always below 0.7%). In this case, the FM and AFM configurations have been computed in order to evaluate their effective pair exchange coupling and thus estimate the Curie temperature. Here, the internal charge transfer is not expected because the 2p energy levels of the two N dopants are degenerate and lie just below the top of the O 2p valence band. The two dopants have almost identical geometrical and electronic environment (see Fig. 2 and Supporting Information), hence the change transfer is energetically not favorable.

We discuss now whether this doped material can exhibit room temperature p-ferromagnetism or not. Long-range ferromagnetism order is possible only if the effective pair exchange interactions between atoms carrying magnetic moments are sufficiently large and extended. Indeed, Curie temperature, T_c_, is usually well below room temperature because of the short-range magnetic exchange interaction of deep impurity states in wide band gap semiconductors. Here, the double exchange is the mechanism that dominates the magnetic properties of the system, as observed in other cases^53^,^54^. The double exchange is indeed predominant for wide band gap semiconductors (e.g. GaN and ZnO) and it is very short ranged due to the exponential decay of the impurity wave function in the gap. The T_c_ is then expected to be low since, due to the short-range interaction, percolation of the ferromagnetic coupling is difficult to achieve for small dopants concentration.

By using the Heisenberg mean field model, T_c_ can be estimated as:


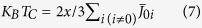


where the sum extends over all sites occupied by magnetic atoms, k_B_ is the Boltzmann constant and x is the magnetic dopant concentration^55^,^56^. However, this approximation tends to highly overestimate the T_c_, as widely reported in literature^57^,^58^. The simplest way to include the crucial effect of the dilution and obtain reliable T_c_ values has been proposed by Maká F. *et al*.^56^. Here, the term 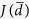, which corresponds to the magnetic coupling at an average distance 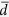 between induced magnetic moments, is computed for a given concentration. 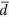 is easily estimated as:


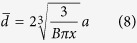


where *a* is the lattice parameter. This leads to:


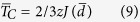


where z is the number of neighbors in the spherical shell defined by the radius 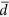. 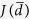 can be easily computed as the difference of the total energy between parallel and antiparallel spin orientation (FM-AFM)^55^.

As reported in Table 1, the FM configuration of 2N_sub3c_-ZrO_2_, where the N—N distance is lower than 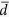, is slightly more stable than the AFM by 6 meV. This value leads to a T_c_ computed according to eq. (9) significantly below room temperature (around 45 K), although the N—N distance does not allow to include the dilution effect (i.e. z = 1). For matter of completeness, the calculation has, therefore, been performed also with the two magnetic dopants placed approximately at the distance 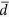, in order to correctly apply equation (9) and to include the dilution contributions. Once this is done, the magnetic coupling 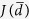 falls to 0.7 meV, thus strongly decreasing T_c_ that becomes 22 K. Therefore, we can exclude room temperature ferromagnetism for this system, differently from what has been suggested in ref. 34. This is a direct consequence of the strong localization of the unpaired electron in the N 2p states. Such a localization is found also at the GGA level of theory, but here the wave function of the magnetic impurity is spatially more extended, resulting in a much larger 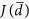 value.

Thus far, we have discussed the magnetic properties assuming that the N dopants are homogeneously distributed. However, it is worth noting that an inhomogeneous distribution can be energetically favorable; in these cases the system can have the potential to give rise to spinodal decomposition. However, for low dopant concentration (as in this case), the spinodal decomposition has the effect to suppress the Curie temperature because it leads to the formation of small clusters of dopants, which are separated by large distances. The interaction between these clusters is therefore negligible, due to the short-range nature of the magnetic interaction of this system. On the contrary, if the clusters are large enough, a superparamagnetic blocking phenomenon becomes important and affects the magnetization process^26^,^59^,^60^,^61^,^62^.

N_sub3c_/N_sub4c_-ZrO_2_ and 2N_sub4c_-ZrO_2_: Internal Charge Transfer: In the second model, N_sub3c_/N_sub4c_-ZrO_2_, an O_3c_ and an O_4c_ have been substituted by two N atoms. The two N impurities are no longer equivalent, and an internal charge transfer can occur, thus forming, besides the FM and AFM configurations, also the ^1^CT and ^3^CT ones. The N—N distance changes are slightly more pronounced, in fact, for ^3^CT and ^1^CT, we can observe an elongation of about 3% with respect to the starting O_3c_—O_4c_ distance (4.4 Å).

Considering the FM and AFM phases, the 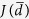 value obtained is still too small (2 meV) to lead to stable magnetic ordering at room temperature, as observed for the 2N_sub3c_-ZrO_2_ model. In addition, the formation of the ^1^CT and ^3^CT configurations, that quenches the magnetic moments of both dopant species, is definitely more stable than the FM one.

The occurrence of an internal charge transfer is due to the different position of the N 2p states of the two N dopants. This, in turns, is a consequence of the different coordination number in N_sub3c_- and N_sub4c_. The energetically lower and more stable N_sub4c_ 2p states are ready to accept an electron from N_sub3c_ states leading to a pair of formally N_sub4c_^−^ and N_sub3c_^+^ species. As we mentioned above, N_sub3c_^+^ can exist in two spin configurations, triplet or singlet, but the magnetic interaction changes completely its nature. This mechanism is further confirmed by the Mulliken charges and by the spin densities of the two N atoms, as shown in Table 2. Considering the ^3^CT configuration, N_sub3c_ exhibits a lower charge with respect to N_sub4c_ (7.62 vs 8.41) and spin density of 1.77, in agreement with the presence of two unpaired electrons on one single dopant.

**Figure 5:**
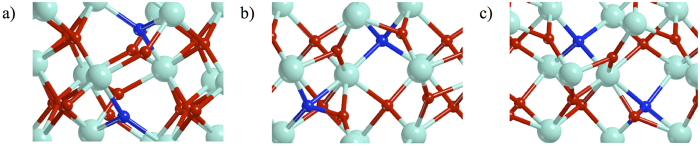
Optimized structures of the most stable magnetic configurations for each 2N-ZrO_2_ model. (**a**) 2N_sub3c_-ZrO_2_ FM; (**b**) N_sub3c_/N_sub4c_-ZrO_2_^1^CT and (**c**) 2N_sub4c_-ZrO_2_^1^CT.

The formation of the diamagnetic phase (^1^CT) is accompanied by an important geometric rearrangement. This consists in the formation of a direct N—O bond (d(N—O) = 1.51 Å). In fact, the O_3c_ nearby to N_sub3c_ dopant, during the optimization process, spontaneously migrates towards the N_sub3c_, thus breaking a Zr—O bond and leaving its lattice position (see Fig. 5). This process can also be seen as the formation of an O vacancy together with an O interstitial, which corresponds to the creation of a Frenkel defect. A detailed discussion on the role of the vacancies in the stabilization of the doped structure is reported in the next subsection.

As mentioned above, the ^1^CT configuration is the most stable one. In particular, it is more stable than the FM phase by 649 meV (Table 2). It is also 417 meV more stable than the FM 2N_sub3c_-ZrO_2_ structure (the most stable configuration of the first model). The occurrence of an internal charge transfer is thus a very efficient mechanism to reduce the number of paramagnetic centers and to quench magnetic ordering (higher dilution).

For completeness, also the 2N_sub4c_-ZrO_2_ has been modeled in order to verify the previous considerations. In this case, the N 2p levels have the same energy for both the dopants, but their slightly different hybridization with the O 2p states still allows the charge transfer thus forming a stable ^1^CT phase. This diamagnetic configuration is generated by a charge transfer from the N_sub4c_—O species, resulting from the optimization processes, to the other tetra-coordinated N dopant, as observed for N_sub3c_/ N_sub4c_ -ZrO_2_. Also in this case, ^1^CT is more stable than the FM by 281 meV (Table 2).

The Role of the Vacancies: We have seen above that during the optimization process of the second and third model, N_sub3c_/N_sub4c_-ZrO_2_ and 2N_sub4c_-ZrO_2_, a migration of the O nearby to an N dopant has taken place, with formation of a N—O species. This migration generates a pseudo-oxygen vacancy that forms spontaneously as a consequence of the internal charge transfer. This result opens the general question of the interplay between N-dopants and formation of O vacancies (V_o_) in the oxide. Previous work on N-doped TiO_2_ has shown that the presence of the N dopants leads to a strong reduction of the cost of O vacancies formation. This is due to the fact that in TiO_2_ an O vacancy is associated with the formation of two Ti^3+^ ions with the 3d electron lying 0.8–1.0 eV below the conduction band. Since a N-dopant introduces hole states in the mid of the gap, the electron transfer from Ti^3+^ to the N impurity allows for a net energy gain that reduces the cost to create the vacancy^63^. A similar phenomenon is expected here for ZrO_2_ since the O vacancy in this material forms a gap state much higher in energy than the N 2p empty level. The formation energy of V_o_ for pure ZrO_2_ is of about 7.2 eV (computed with respect to 1/2 O_2_), thus confirming the non-reducible nature of ZrO_2_^64^. With such a high formation energy, the number of these defects at thermodynamic equilibrium for the pure oxide is not expected to be high. However, if two N dopants are introduced in the model, a reduction of the V_o_ formation energy down to 1.9 eV for N_sub3c_/N_sub4c_-ZrO_2_ is shown. This is a very large change in the cost of formation of a neutral V_o_ (about 5 eV). The vacancies are therefore much more easily formed in systems containing dopants.

Therefore, the presence an acceptor species (tetra-coordinated N atoms with deep 2p states hybridized with O 2p states), in combination with a donor species, facilitates the vacancy formation that in turn leads to a stabilization of the system in a diamagnetic phase, thus quenching the magnetic moment of the N-impurities. The p-ferromagnetism in this system is then unlikely due to the reduced number of magnetic impurity centers.

## Summary and Conclusions

In this work we have studied the nature of isolated N-dopants in the bulk of ZrO_2_ and their magnetic interaction. The topic of diluted magnetic seminconductors has attracted a considerable interest in the past, but the theoretical description of these systems requires accurate methods able to properly describe the degree of spin localization in magnetic defects. To this end, we have performed DFT calculations using the self-interaction corrected B3LYP hybrid functional which gives an acceptable description of the Kohn-Sham band gap of the pristine material. We have considered both substitutional to O and interstitial nitrogen dopants. Since in ZrO_2_ there are both O_3c_ and O_4c_ ions, N-atoms have been introduced in the corresponding O_3c_ and O_4c_ positions.

A thermodynamic analysis of the stability of the various doping situations as a function of the oxygen partial pressure shows that at 300 K, N is incorporated in interstitial sites at any range of oxygen pressure. At higher temperatures, typical of the annealing process in the synthesis of oxides, oxygen poor-conditions facilitate the formation of substitutional dopants.

Both substitutional N-species exhibit a singly occupied N 2p level; interstitial nitrogen, on the other hand, tends to bind to a lattice O atom with formation of an N-O species, an effect that has been found in several other N-doped oxides. In this case, the unpaired electron occupies a level which is reminiscent of a π MO in the NO gas-phase molecule. A feature common to both substitutional and interstitial nitrogen is that the unpaired electron is rather localized, as proved by the calculation of the hyperfine coupling constants of the electron spin with the N nuclear spin.

The fact that the nitrogen dopant introduces magnetic states in the material has previously led to the suggestion that N-ZrO_2_ can behave as a diluted magnetic semiconductor exhibiting ferromagnetic ordering at room temperature^34^. Some calculations, performed with standard GGA DFT functional, which however suffers from self-interaction problems, were proposed to prove it. Here we have considered the interaction of two N defects in the same supercell. Both ferro- and anti-ferromagnetic orderings have been calculated, considering dopants in various positions, and changing their distances. In all cases we found very small magnetic coupling constants J(d), such that magnetic ordering can be achieved only at very low temperatures, well below liquid nitrogen, ruling out the possibility that magnetic ordering can be achieved at room temperature with this kind of doping.

There is another more relevant reason why room temperature ferromagnetism is unlikely in N-ZrO_2_. The calculations clearly show a tendency, observed already in other materials, to internal charge transfers. If N atoms replace O at different sites, thus resulting in slightly different position of the corresponding N 2p levels, a direct charge transfer can occur:


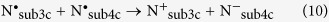


N^+^_sub3c_ (2p^4^) can exist in singlet or triplet configuration, but the magnetic moment is completely quenched in N^−^_sub4c_, which has completely filled 2p levels. This reduces the concentration of magnetic impurities.

A similar mechanism that leads to complete magnetic quenching involves the formation of oxygen vacancies. These defects have very high formation energies in pure ZrO_2_, but can form at a much lower cost in the N-doped phase. In this case the process is the following:


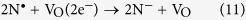


Here, the two electrons associated to an O vacancy, which occupy energy levels lying high in the gap, are transferred to the low-lying N 2p (or 2π) levels, with a consequent net energy gain. This mechanism leads to a complete quenching of the magnetic moment on the N-dopants, and further contributes to the reduced magnetic ordering at high temperature.

## Additional Information

**How to cite this article**: Albanese, E. *et al*. Magnetic properties of nitrogen-doped ZrO_2_: Theoretical evidence of absence of room temperature ferromagnetism. *Sci. Rep.* **6**, 31435; doi: 10.1038/srep31435 (2016).

## Supplementary Material

Supplementary Information

## Figures and Tables

**Table 1:** Calculated ^14^N Hyperfine Coupling Constants (mT) and Spin Densities (ρ) for the N-ZrO_2_ structures.

	A_1_	A_2_	A_3_	a_iso_	T_1_	T_2_	T_3_	Ρ
N_sub3c_-ZrO_2_	2.841	−0.780	−0.806	0.418	2.422	−1.198	−1.224	0.92
N_sub4c_-ZrO_2_	2.538	−0.743	−0.726	0.356	2.182	−1.082	−1.099	0.86
N_int3c_-ZrO_2_	3.563	−0.472	−0.612	0.826	2.737	−1.299	−1.438	0.84
N_int4c_-ZrO_2_	3.523	−0.343	−0.449	0.910	2.613	−1.253	−1.360	0.80

**Table 2:** N-N distance (Å), Mulliken charges and spin densities for each N dopant, relative stabilities (meV) with respect to each model (ΔE_rel_) and the total one (ΔE_tot_).

	d(N-N)		N1		N2		ΔE_rel_	ΔE_tot_
			Charge	ρ	Charge	ρ		
			N_sub3c_		N_sub3c_			
2N_sub3c_-ZrO_2_	3.826	FM	7.971	0.916	7.972	0.916	0	+417
	3.824	AFM	7.971	0.918	7.971	−0.918	+6	
			N_sub4c_		N_sub3c_			
N_sub3c_/N_sub4c_-ZrO_2_	4.278	FM	8.102	0.854	7.975	0.918	+649	
	4.279	AFM	8.102	0.849	7.975	−0.918	+647	
	4.572	^3^CT	8.411	0.036	7.625	1.773	+220	
	4.402	^1^CT	8.443		7.837		0	0
			N_sub4c_		N_sub4c_			
2N_sub4c_-ZrO_2_	4.110	FM	8.108	0.853	8.106	0.851	+544	
	4.030	^1^CT	8.462		7.957		0	+281
