# Supplementary material for: Magnetic properties of nitrogen-doped ZrO2: Theoretical evidence of absence of room temperature ferromagnetism
Source: Sci Rep. 2016 Aug 16;6:31435. doi: 10.1038/srep31435 (PMC4985629; doi:10.1038/srep31435)

Magnetic properties of nitrogen-doped ZrO_2: Theoretical evidence of absence of room temperature ferromagnetism

Elisa Albanese, Mirko Leccese, Cristiana Di Valentin, and Gianfranco Pacchioni

Dipartimento di Scienza dei Materiali,

Università Milano Bicocca, via R. Cozzi 55, 20125 Milano, Italy

Table S1: Mean cell parameters of all the singly N-doped structures (Å and degree).

	a	b	c	α	β	γ
ZrO_2	5.240	5.265	5.417	90	99.43	90
N_{sub3c}-ZrO_2	5.248	5.267	5.418	89.88	99.51	90.10
N_{sub4c}-ZrO_2	5.241	5.268	5.426	90.02	99.37	89.93
N_{int3c}-ZrO_2	5.244	5.267	5.432	90.35	99.12	89.70
N_{int4c}-ZrO_2	5.246	5.292	5.450	90.62	99.54	89.78

Figure S1: Total and Projected Densities of States of $2N_{sub3c}$-ZrO_2 in ferromagnetic and antiferromagnetic configurations. The insets show the spin density plots (isodensity threshold values 0.007). The Fermi level is set to the highest occupied level (dashed line).

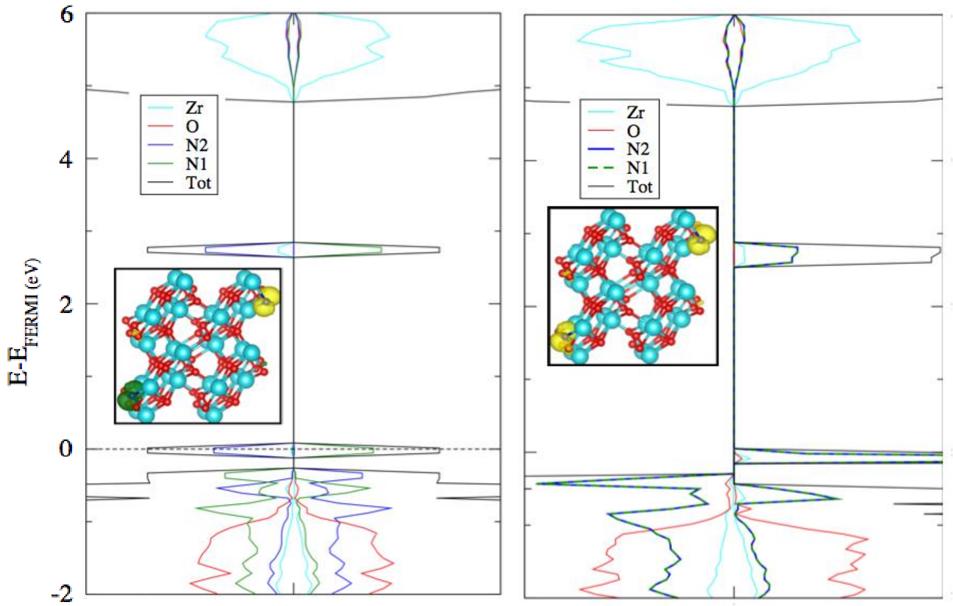

Figure S2: Total and Projected Densities of States of N_{sub3c}/N_{sub4c} -ZrO_2 in ferromagnetic and antiferromagnetic configurations. The insets show the spin density plots (isodensity threshold values 0.007). The Fermi level is set to the highest occupied level (dashed line).

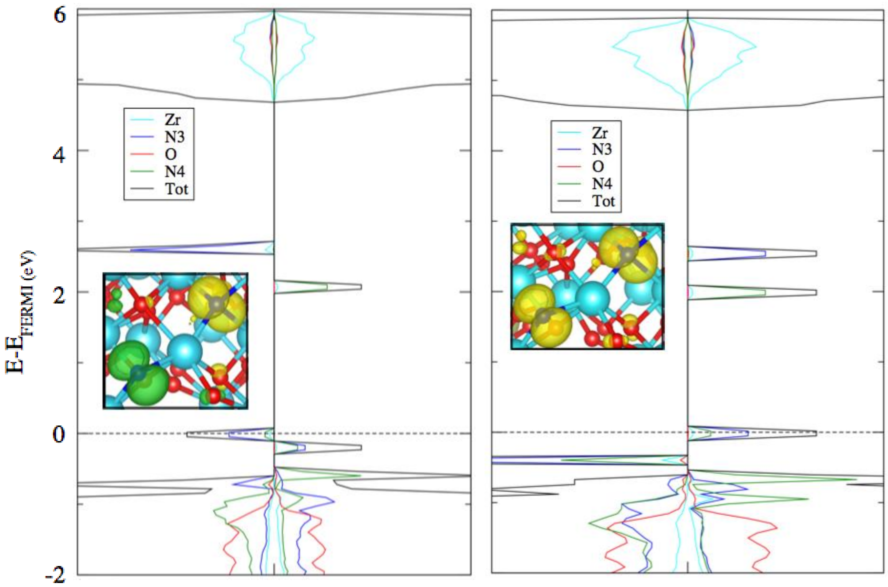

Figure S3: Total and Projected Densities of States of $2N_{sub4c}$ -ZrO_2 in the ferromagnetic configuration. The inset shows the spin density plot (isodensity threshold values 0.007). The Fermi level is set to the highest occupied level (dashed line).

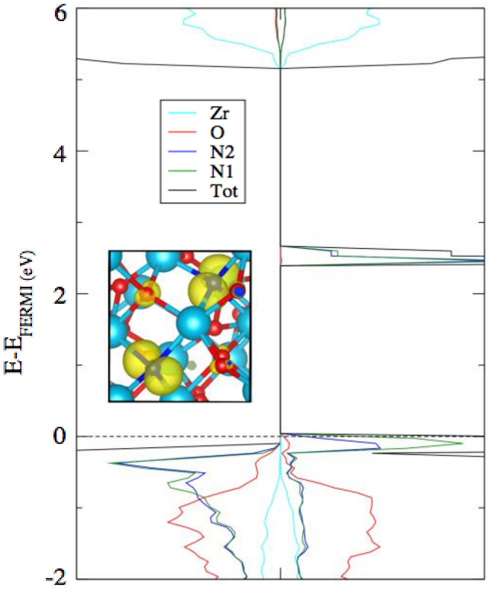

Supplement: Supplementary Information [file srep31435-s1.pdf]
